# Immunology's Coming of Age

**DOI:** 10.3389/fimmu.2019.00684

**Published:** 2019-04-03

**Authors:** Stefan H. E. Kaufmann

**Affiliations:** ^1^Department of Immunology, Max Planck Institute for Infection Biology, Berlin, Germany; ^2^Hagler Institute for Advanced Study, Texas A&M University, College Station, TX, United States

**Keywords:** antibody, cytokine, dendritic cell, immunology, lymphocyte, macrophage, phagocytosis, recombination

## Abstract

This treatise describes the development of immunology as a scientific discipline with a focus on its foundation. Toward the end of the nineteenth century, the study of immunology was founded with the discoveries of phagocytosis by Elias Metchnikoff, as well as by Emil Behring's and Paul Ehrlich's discovery of neutralizing antibodies. These seminal studies were followed by the discoveries of bacteriolysis by complement and of opsonization by antibodies, which provided first evidence for cooperation between acquired and innate immunity. In the years that followed, light was shed on the pathogenic corollary of the immune response, describing different types of hypersensitivity. Subsequently, immunochemistry dominated the field, leading to the revelation of the chemical structure of antibodies in the 1960s. Immunobiology was preceded by transplantation biology, which laid the ground for the genetic basis of acquired immunity. With the identification of antibody producers as B lymphocytes and the discovery of T lymphocytes as regulators of acquired immunity, lymphocytes moved into the center of immunologic research. T cells were shown to be genetically restricted and to regulate different leukocyte populations, including B cells and professional phagocytes. The discovery of dendritic cells as major antigen-presenting cells and their surface expression of pattern recognition receptors revealed the mechanisms by which innate immunity instructs acquired immunity. Genetic analysis provided in-depth insights into the generation of antibody diversity by recombination, which in principle was shown to underlie diversity of the T cell receptor, as well. The invention of monoclonal antibodies not only provided ultimate proof for the unique antigen specificity of the antibody-producing plasma cell, it also paved the way for a new era of immunotherapy. Emil Behring demonstrated cure of infectious disease by serum therapy, illustrating how clinical studies can stimulate basic research. The recent discovery of checkpoint control for cancer therapy illustrates how clinical application benefits from insights into basic mechanisms. Last not least, perspectives on immunology progressed from a dichotomy between cellular-unspecific innate immunity and humoral-specific acquired immunity, toward the concept of complementary binarity.

## Introduction

**Figure 1:**
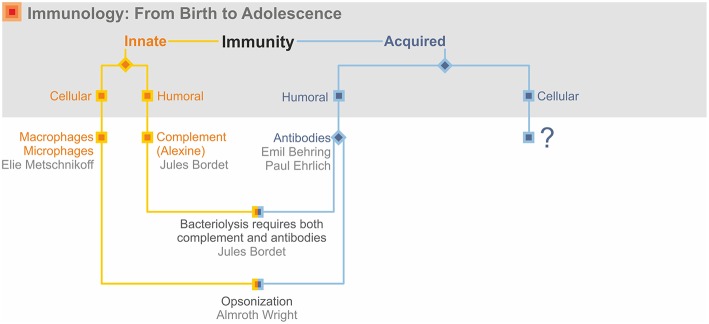
Immunology's early days.

In this treatise, I describe growth and maturation of immunology as a scientific discipline built on both basic research and medical application. Although I emphasize the birth of immunology and early decades of its evolution, I stress that immunology in its full maturity remains equally integrated in both basic and clinical research. Immunology started in the last quarter of the nineteenth century with two major discoveries. The first of these was Elias Metchnikff's (1845–1916) identification of phagocytic cells, which engulf and destroy invading pathogens (1). This laid the basis for innate immunity. The second discovery was Emil Behring's (1854–1917) and Paul Ehrlich's (1854–1915) identification of antibodies, which neutralize microbial toxins (1, 2). This became the basis for acquired immunity. These findings also led to the distinction between cellular and humoral immunity. For obvious reasons, humoral immunity was often considered synonymous with acquired immunity, whereas cells were considered tightly linked to innate immunity. This was overlaid by a further segregation between the unique antigen specificity of the acquired arm vs. the non-specific innate arm of the immune response (Figure 1). This dichotomous view led to some confusion and controversy and it took some time until it transformed into a perspective of complementary binarity considering innate and acquired immunity as interactive partners. Today the two arms of antigen-specific acquired and antigen-nonspecific innate immunity are best viewed as a ying–yang concept, with highly intertwined, partly overlapping, and mutually beneficial activities. Further highly valuable information on the highlights of immunology in its nascence can be found in the many publications of A. Silverstein of which I only cite his major treatise (3).

From its birth, immunology was at the heart of biomedical research providing both crucial information on basic biological processes and on clinical application. This was recognized by the first ever Nobel Prize in Medicine awarded in 1901 to Emil Behring “for serum therapy in therapeutic medical science,” (4) and also by the most recent Nobel Prize 2018 to honor the “discovery of cancer therapy by inhibition of negative immune regulation” by Jim Allison (1948–) and Tasuku Honjo (1942–) (5). Whilst Behring's discovery illustrates how medical application can stimulate basic research, the discoveries of Allison and Honjo epitomize clinical application as the result of in-depth understanding of basic biological mechanisms.

## Act I: The Foundation of Immunology

Immunology emerged as an academic discipline in its own right out of the fertile soil of medical microbiology (6). The discoveries of Louis Pasteur (1822–1895), which confirmed and completed the germ theory of infectious diseases as well as Robert Koch's (1843–1910) meticulous studies on the etiology of infectious diseases, notably tuberculosis, raised a question of fundamental importance: Is the host a helpless prey of pathogenic microbes or is it equipped with an efficient defense mechanism to combat its invaders? Both Pasteur and Koch favored the notion that the host was defenseless. However it was Metchnikoff, at the Pasteur Institute in Paris since 1888, who earlier discovered the critical role of phagocytosis and intracellular killing in host defense (1), and it was Behring and Ehrlich, young independent researchers at Koch's institute for Infectious Diseases in Berlin, who identified antibodies as crucial counterparts to the toxic activities of bacteria (1, 2). We now know that the outcome of infection depends on close interactions between pathogen and host factors, probably best described by the term infection biology.

When Koch embarked on the next step in his career in Berlin in 1878, the pathologist Rudolf Virchow (1821–1902) was the most eminent professor at the Charité clinics (6). Virchow is the founder of cellular pathology, which assumes that all diseases are the result of malfunctioning of our body's cells (7). Hence, Koch's ideas on the etiology of infectious diseases seconded by the germ theory of Pasteur were highly criticized by Virchow. Ultimately, Koch's observations, well-supported by experimental evidence, became the accepted paradigm. According to the American physicist and philosopher, Thomas Kuhn (1922–1996), normal science progresses as long as available evidence can be accommodated in the existing paradigm (8). Once anomalies accumulate from scientific research that can no longer be integrated in an existing paradigm, the time is ripe for a paradigm shift (8). Koch and Pasteur introduced a paradigm shift by demonstrating that exogenous invaders can cause certain diseases, beyond those diseases caused by dysfunctional cells. Yet, they both largely overlooked the role of host immunity as important defense mechanism. This paradigm shift was initiated by Metchnikoff, Behring, and Ehrlich. Today we understand infectious diseases as the outcome of a crosstalk between host and pathogen. We also now know that immunology has more roles to play than only pathogen defense, such as surveillance of malignant cells. Moreover, a dysfunctional immune system results in allergy, autoimmunity or chronic inflammation thereby illustrating it as a double-edged sword.

### Phagocytosis

Metchnikoff was born in 1845 in a part of Russia, which now belongs to the Ukraine (9). He studied zoology and soon became a traveling scientist. Notably, when working at the Zoological Station in Naples he studied simple organisms and identified specialized cells dedicated to nutrient uptake. These nutrients could be contained in particles and thus the concept of phagocytosis was conceived as a process of uptake of particles or microbes rich in food. Moreover, in his experiments with starfish larvae in Messina in 1883, Metchnikoff found that phagocytic cells were highly motile and migrated to sites of foreign insult (10). He later wrote about these groundbreaking observations:

“… I fetched from it a few rose thorns and introduced them at once under the skin of some beautiful starfish larvae as transparent as water. I was too excited to sleep that night in the expectation of the result of my experiment and very early the next morning I ascertained that it had fully succeeded. That experiment formed the basis of phagocyte theory to the development of which I devoted the next 25 years of my life …” (11).

**Figure 2:**
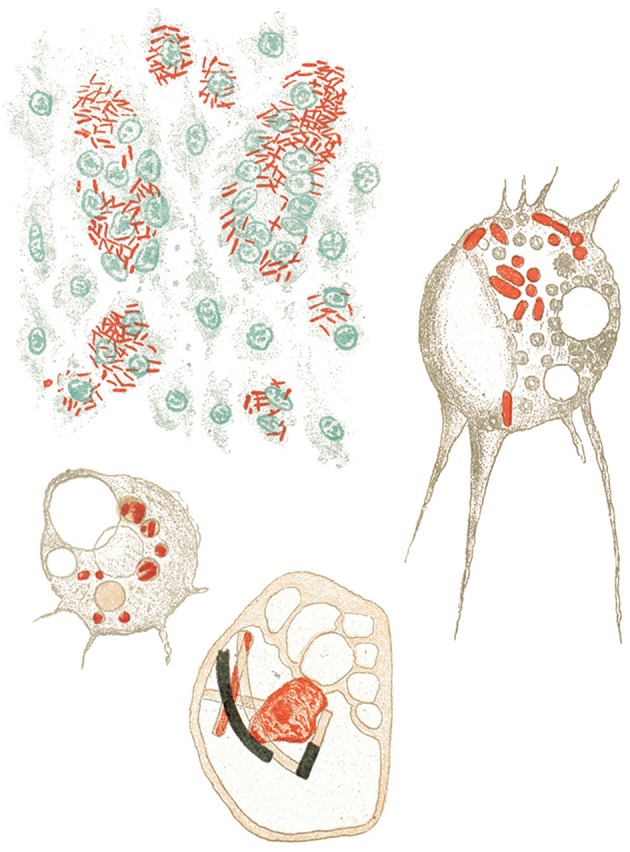
Metchnikoff's view on phagocytosis of different bacterial pathogens (12).

Indeed, Metchnikoff changed his scientific interests from zoology to pathology and in this way became one of the first immunologists. He discovered phagocytes in vertebrates and began analyzing phagocyte functions in infectious diseases, such as anthrax, sepsis, and tuberculosis (Figure 2). Based on these studies, he distinguished macrophages from microphages (which we now call neutrophils) according to the form of their nucleus:

“… I suggest calling all elements macrophages, which generally possess a simple non-polymorphic nucleus that is round or frequently oval. … as microphages I call smaller amoeboid cells, which can be easily stained, with a largely polynuclear and fragmented nucleus and faint protoplasm….” (13).

### Serum Therapy and Antibodies

**Figure 3:**
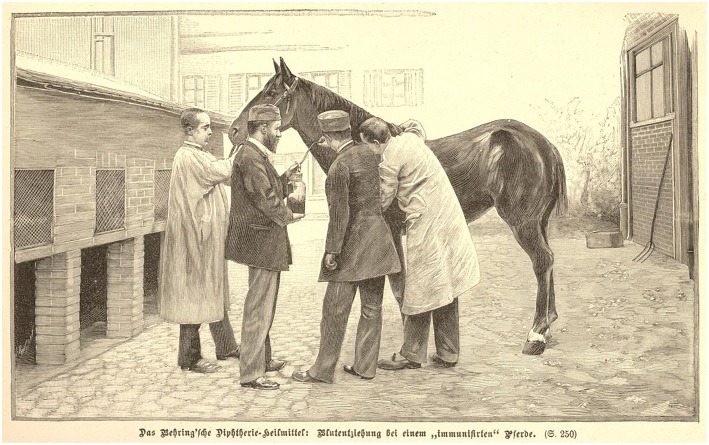
Large-scale production of serum against diphtheria toxin.

Behring was born in the German province of Prussia, now part of Poland, in 1854 (14). He studied medicine at an army academy and soon became interested in studies on the curative activity of disinfectants in bacterial infections. During his experiments on antiseptic activity of small molecules, together with the Japanese guest researcher Shibasaburo Kitasato (1853–1931) at the Institute for Infectious Diseases in Berlin, he discovered that serum from infected animals contained antibacterial activity that was specific for the infectious agent (15). Essentially, the activity was directed against the bacterial toxin. Whilst the joint paper of Behring and Kitasato mostly focused on tetanus and its toxin, the single-authored paper by Behring published shortly thereafter, described protection against diphtheria and its toxin by antisera (15, 16). Soon these animal experiments were translated into a human study, which revealed that serum therapy protected against diphtheria when given during early stages of infection or even during disease. Behring joined forces with industry to produce large doses of antisera for human use, thus embodying the translational immunologist with great interest in medical application (Figure 3). His serum therapy was a breakthrough and honored by the first ever awarded Nobel Prize in Medicine in 1901 (4).

Serum therapy was more than just a curative method. It also provided supportive evidence for the idea that the cause of infectious disease is highly specific and that this specificity is linked to toxins produced by the etiologic pathogen. As a corollary, the cure of the specific disease was accompanied by a specific poison-averting (antitoxic) agent, which circulates in blood and can offer specific protection against the toxin in other individuals (15–17).

Despite all the honors he received, Behring was not fully satisfied with passive vaccination. It took him some 20 years to solve the issue of active vaccination (18). In 1913, at the Congress for International Medicine in Wiesbaden, Germany, Behring gave a remarkable presentation, which the newspaper “Vossische Zeitung” (April 18, 1913) described quite aptly:

“At today's discussions, Behring appeared as lively as ever and reported on a new protective agent comprising a mixture of diphtheria toxin and anti-toxin. This agent was harnessed for treating individuals at risk prophylactically. It was found that first the agent was completely innocuous, and second that the appearance of true protection could be demonstrated by the formation of sufficiently high abundance of protective agents in the blood of immunized individuals who all remained free of diphtheria” (14).

In order to neutralize the diphtheria toxin, Behring generated antigen-antibody complexes, which stimulated production of toxin-specific antibodies in the immunized host. This was an important, but still suboptimal start toward active vaccination against bacterial toxins. It was the French researcher, Gaston Ramon (1886–1963), who ultimately introduced detoxification by formaldehyde for low-cost production of safe vaccines against diphtheria and tetanus, and aluminum hydroxide as adjuvant for potent immunization (19, 20).

Whilst Behring was a translational immunologist, who contributed significantly to basic immunology, Ehrlich was most interested in the in-depth understanding of basic mechanisms underlying immunity, and contributed profoundly to the clinical development of serum therapy. Indeed it was Ehrlich whose contribution made large-scale production of antisera of reproducible quality possible. By working out “a new and more accurate method for determining the value of the serum and to study the complex relations which govern the neutralization of toxin and antitoxin,” he could show that “… the immunity unit is no longer an arbitrary concept, but is an exactly determinable quantity and one therefore which can be reproduced afresh at any time …” (21). Ehrlich was therefore the first to provide the basis for a quality control measure of a biological. At those times, this was urgently needed because of widespread state-controlled compulsory vaccination against smallpox.

**Figure 4:**
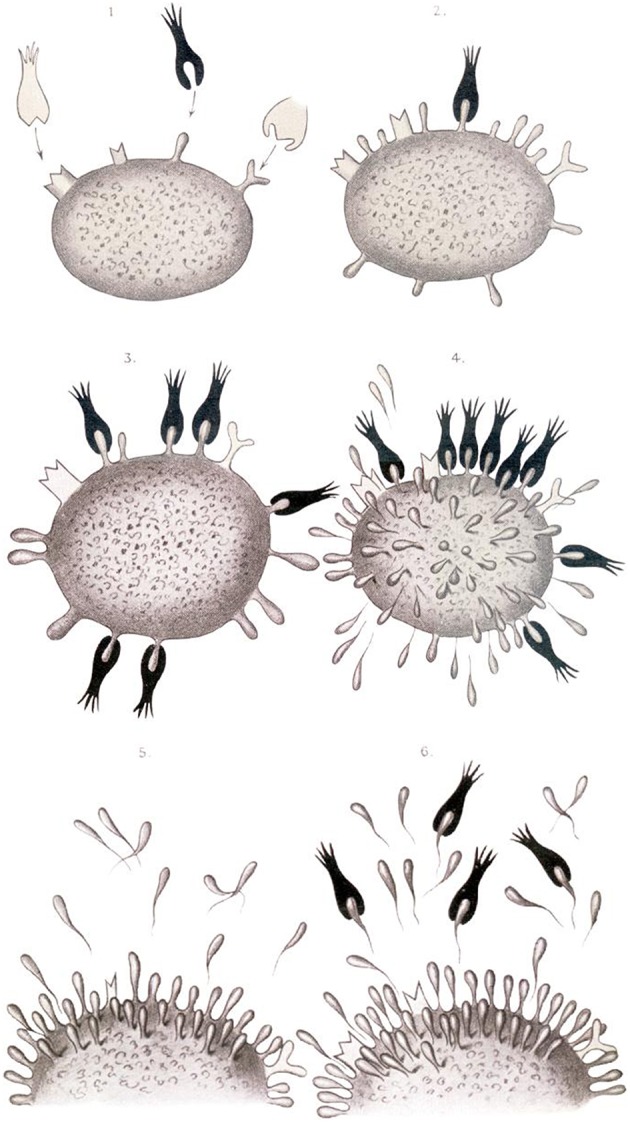
Ehrlich's view on antibody secretion to different antigens (23).

Yet, Ehrlich became most famous for basic research of, and stimulating ideas on, how the immune system works. In his MD thesis, Ehrlich described mast cells which, as we now know, are critical effectors of allergy (22). But his most important findings are related to antibodies. He foresaw that antigens, such as toxins, stimulate the production of specific antibodies. Interestingly, similar to Metchnikoff, Ehrlich assumed a nutritional point of view (22). Different cells need different kinds of nutrients and hence Ehrlich postulated specific receptors as being responsible for nutrient uptake. From this he concluded that the cell receptor specific for a given toxin should fulfill similar criteria. Because of the sheer abundance of toxins generated during infection, more specific receptors are produced and are ultimately secreted into the serum (Figure 4). In the Croonian Lecture given in 1900 at the Royal Society, Ehrlich reflected on his ideas as follows:

“… the first stage in the toxic action must be regarded as being the union of the toxin …. to a special side chain of the cell protoplasm. … the side chain involved, so long as the union lasts cannot exercise its normal physiological nutritive function. …. such an excess of side chains is produced that to use a trivial expression, the side chains are present in too great quantity for the cell to carry and are, after a manner of secretion, handed over as superfluous ballast to the blood …” (23).

Essentially this is the core message of the side chain theory for which Ehrlich is most renowned. But Ehrlich was far more productive. He showed that the milk of breastfeeding mothers carries antibodies beneficial to the suckling infant, thus providing the child with a high degree of immunity (24). He speculated on the role of tolerance to self and the risk of autoimmunity and coined the well-known term “horror autotoxicus” (24). He revealed several biological features of complement, which was originally discovered by the German scientist, Hans Buchner (1850–1902), and the Belgian researcher, Jules Bordet (1870–1961), who termed it alexine (25). Ultimately, however, the term complement created by Ehrlich prevailed. Bordet and Buchner had already shown that alexine was heat-labile (25–27). Buchner used serum from non-immunized animals, whereas Bordet included serum from immunized animals in his studies and so distinguished the heat-labile alexine from the heat-stable antibodies. Ehrlich, together with his colleague Richard Pfeiffer (1858–1945), further defined the activities of antibodies and complement by mixing untreated and heat-inactivated serum. In his own words, Ehrlich summarized this finding: “The two substances are (i) the specific immune body produced by immunization and (ii) a substance which usually is thermo-labile, contained even in normal serum” (28).

In 1908 Ehrlich and Metchnikoff were jointly awarded the Nobel Prize in Physiology or Medicine “in recognition of their work on immunity” (29). Bordet was honored “for his discoveries relating to immunity” with the Nobel Prize in 1919 (30).

The interaction of complement and antibodies was the first dent in the dichotomous view of immunity (Figure 1). Complement was part of the innate immune response and hence non-specific. But it was humoral. Thus, the exclusive association of innate immunity with cells had become obsolete. More importantly, specific antibodies cooperated with non-specific complement.

The dichotomous view of immunology was further softened by the experiments of the English scientist, Almroth Wright (1861–1947), who showed that antibodies can specifically facilitate phagocytosis of bacteria (31, 32). This is of particular importance for efficient defense against bacterial pathogens which evade phagocytosis, such as encapsulated bacteria (pneumococci, meningococci and gonococci). His finding revealed that for some diseases, specific antibodies are needed to interact with phagocytes for optimal host defense (31, 32). For the first time therefore, specific humoral factors of the acquired immune response (antibodies) were shown to collaborate with non-specific cognates of the cellular innate immune response (macrophage and neutrophils). This was another call for complementary dualism rather than dichotomy between innate and acquired immunity. The findings of Wright caught the interest of George Bernard Shaw (1856–1950), who described the potential of phagocytes for cellular therapy of disease. In Act I of “The Doctor's Dilemma,” he writes: “There is at bottom only one genuinely scientific treatment for all diseases and that is to stimulate the phagocytes.” During the play, however, the risk of adverse events of such therapy is increasingly recognized and culminates in the question: “Have we overstimulated the phagocytes? Have they not only eaten up the bacilli but attacked and destroyed the red corpuscles, as well?” Adoptive phagocyte therapy never made it into the clinics as an immunologic treatment regimen.

## Act II: Immunochemistry and Clinical Immunology

### Immunochemistry

During the first half of the twentieth century, immunologists focused on clinical observations and even more on immunochemistry, which could build on a much broader armamentarium of technical tools. Immunochemistry found its culmination in the discovery of the chemical structure of antibodies (Figure 4). This was accomplished independently by the British chemist, Rodney Porter (1917–1985), and the US chemist, Gerald Edelman (1929–2014), in the late 1950s to early 1960s (33, 34). Their work was honored by the Nobel Prize in 1972 (35). The Austrian Karl Landsteiner (1868–1943), first working in Europe and since 1923 in the US, developed the carrier hapten concept by coupling small aromatic molecules to proteins (36). He showed that the small residue—the hapten—is recognized by antibodies, and therefore serves as epitope, and that the protein serves as carrier to provide the immunogenicity needed for successful stimulation of an antibody response (37, 38). Since the studies of Jacques Miller (1931–), Henry Claman (1930–2016) and others, we know that the antibody response involves B lymphocytes for the recognition of the hapten and T lymphocytes for the recognition of the carrier.

### Hypersensitivity Reactions

Landsteiner is probably best known for the discovery of the ABO major blood group system (39). Working at the time in Vienna, he found that mixing blood of two different individuals resulted in clumping of red blood cells. Based on this finding, he developed a technique for the serologic differentiation of erythrocytes, which allowed him to identify the different blood groups of the ABO system. This discovery was honored by the Nobel Prize in 1930 (40). Ten years later, and together with Alexander Wiener (1907–1976), Landsteiner discovered a second important blood group, called Rhesus (Rh), named after their original discovery with erythrocytes in Rhesus monkeys (41, 42).

Landsteiner's discovery of so-called isoagglutinins—the antibodies responsible for clumping of erythrocytes when mixed with serum from a donor of a different ABO blood group—were criticized by Paul Ehrlich who considered this finding contradictory to his proposed “horror autotoxicus.” Yet, increasing evidence arose that horror autotoxicus, i.e., autoimmune attack against host cells or molecules was not an absolute no-go for the immune system. It became clear that antibodies do not only perform beneficial functions. That aberrant antibody responses could lead to hypersensitivity reactions was first shown by the French clinician Charles Richet (1850–1935) in 1902 (43), who was awarded the Nobel Prize for his research on anaphylaxis in 1913 (44). The term anaphylaxis was coined by Richet to describe harmful reactions, which were later shown by the Japanese immunologist Kimishigi Ishizaka (1925–2018) and his wife Teruko (1926–), to be mediated by antibodies of the IgE isotype (45). One year after Richet's discovery, the French researcher, Maurice Arthus (1862–1945), described a similar yet distinct type of reaction which he induced experimentally by local injection of antigen into the skin of an individual previously immunized with the same antigen (46). In contrast to the reaction described by Richet, this one was mediated by immune complexes and involved complement. With serum therapy against diphtheria and tetanus broadly applied, numerous individuals received serum from horses in which the antiserum had been generated. In 1905, the clinicians, Clemens von Pirquet (1874–1929) from Austria, and Béla Schick (1877–1967) from Hungary, together observed that multiple injections of such serum could result in serum sickness due to the formation of immune complexes (47). They termed this type of reaction “allergy,” which has come to be applied in a broader sense. Yet, another hypersensitivity reaction was first observed by the Japanese physician, Hakaru Hashimoto (1881–1934), in 1912 (48): “Hashimoto's thyroiditis” turned out to be an autoimmune disease partially mediated by IgG antibodies, which facilitate damage by phagocytes and NK cells. This type of hypersensitivity is also the basis of erythrocyte damage after blood transfusion, e.g., from ABO-disparate donors. At Rockefeller University, Karl Landsteiner together with the American researcher, Merrill Chase (1905–2004), studied the tuberculin reaction first described by Robert Koch and demonstrated that this reaction can be adoptively transferred by cells of an immune animal but not by serum (49). As we know now, the “delayed-type hypersensitivity” reaction mostly involves T lymphocytes.

The four different types of hypersensitivity were categorized by the UK physicians, Philip Gell (1914–2001), and Robin Coombs (1921–2006), in 1963 (50). In this categorization, type I hypersensitivity is the typical IgE-mediated allergy first described by Richet; type II is IgG plus complement-mediated destruction of host cells; type III is mediated by immune complexes such as the Arthus reaction; and type IV is the delayed-type hypersensitivity reaction, including the tuberculin reaction and contact dermatitis. Hashimoto's thyroiditis, originally considered type II, is now known to be a mix of type II and type IV, i.e., it is antibody- and T cell-mediated.

## Act III: The Rise of Immunobiology

### Transplantation Biology

**Figure 5:**
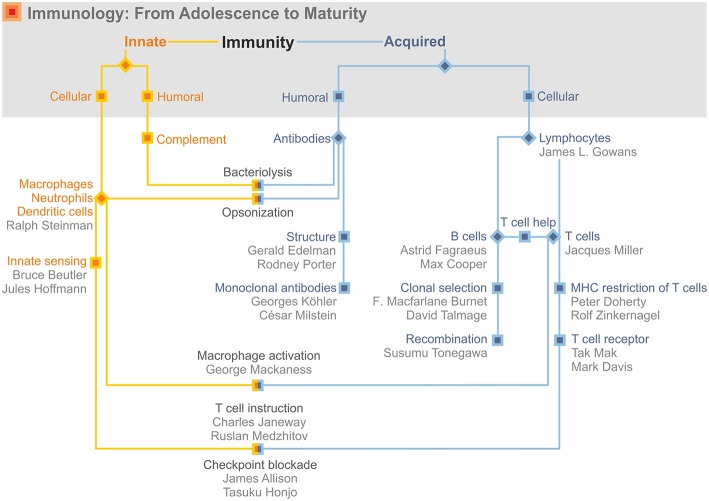
Immunology: from adolescence to adulthood.

The 1950s to 1960s witnessed a marked shift in priorities from immunochemistry to immunobiology (Figure 5). In fact, studies on transplant rejection preceded and prepared the ground for immunobiology. The US geneticist George Snell (1903–1996), based on his studies with inbred mouse strains, elegantly demonstrated that distinct genes within the major histocompatibility complex (MHC) were responsible for transplant rejection (51). The French clinician, Jean Dausset (1916–2009), discovered the human MHC, also named human leukocyte antigen (HLA), on the basis of family studies (51). A somewhat more direct link to immunobiology was provided by the Venezuelan-born US scientist, Baruj Benacerraf (1920–2011), who identified the immune response genes within the MHC locus (51). In 1980, Snell, Dausset and Benacerraf were honored by the Nobel Prize “for their discoveries concerning genetically determined structures on the cell surface that regulate immunological reactions” (52). Later the Australian researcher, Peter Doherty (1940–), and the Swiss researcher, Rolf Zinkernagel (1944–), would broaden this perspective by showing that the MHC is crucial for antigen recognition by T lymphocytes, the cells that would become the dominant research target in the second half of the twentieth century.

### Antibody Specificity Revisited

The Australian virologist, Frank Macarlane Burnet (1899–1985), and the UK biologist, Peter Brian Medawar (1915–1987), received the Nobel Prize in 1960 “for their discovery of acquired immunological tolerance” (53). It was they who provided first evidence that the horror autotoxicus, envisaged by Paul Ehrlich, was not prefixed but a matter of education. Medawar had shown that transplant rejection could be prevented by transferring cells from an unrelated donor during neonatal life (54, 55). Cells from the same donor were later accepted by such mice showing that during fetal and neonatal development the immune system “learned” to accept self. Indeed it was Burnet who outlined the concept of “self vs. non-self” (55). Although his concept remained speculative and was questioned because of the occurrence of autoimmune diseases, it proved to be a valid theory of immunobiology even though—as with many biological issues—it was not absolute. In fact, impact of “self vs. non-self” on immune tolerance remains a matter of controversial discussions—not the least after the realization that self/non-self discrimination is not only a matter of the acquired but also of the innate immune response (see below). Burnet's interests were much broader. Originally a virologist who became an immunologist, he readily used tools of virology to interrogate the immune system. He is probably most famous for postulating the “clonal selection” theory, which again had been triggered by Paul Ehrlich (56). Although Ehrlich's side chain theory held that antibody specificities of all kinds were present before antigen encounter, according to Ehrlich numerous specificities could be expressed by a single cell depending on its requirement for specific nutrients (see Figure 4). This assertion, however, was questioned during the area of immunochemistry when a chemical explanation was sought for a biological question. Several researchers including the US Nobel laureate of 1954 and 1963, Linus Pauling (1901–1994), claimed that the structure of the antigen would determine the specificity of its corresponding antibody (57). In the “template hypothesis,” the antigen binding site was the result of a specific chemical formation around a foreign entity. With the understanding that the three-dimensional structure of a protein is strongly determined by its amino acid sequence, this became a matter of impossibility.

The Danish immunologist, Niels Jerne (1911–1994), who received the Nobel Prize in 1984 (58), postulated a more biologically oriented hypothesis, namely that various antibody specificities existed prior to antigen encounter (59). This was then refined by Burnet and independently by the US immunologist David Talmage (1919–2014), who both proposed a selection process for the specific antibody-producing cell (56, 60). Thus, Ehrlich was right in assuming the preexistence of antibody specificities before a foreign antibody arrived, but he was wrong in assuming that one cell would express numerous specificities. Elegant studies by the Australian immunologist, Gustav Nossal (1931–), partly together with US Nobel laureate of 1958 Joshua Lederberg (1925–2008), provided strong evidence that a single cell produces an antibody of unique specificity (61, 62). Under the influence of the specific antigen, the antibody-producing cells expand numerically and produce more antibodies of the same specificity. Hence, interest in antibodies shifted from chemical structure to biological understanding of the generation of specificity, i.e., on the antibody-producing cell.

### Lymphocytes as Masters of Ceremony

The major cell type of the acquired immune response, however, was still missing (Figure 5). It was the Australian immunologist, Jacques Miller (1931–), who discovered the role of the thymus in the development of a specific lymphocyte population; this finding led to the identification of T lymphocytes as major regulators of the acquired immune response (63). Independent from Miller, the US transplant immunologist Robert Good (1922–2003) characterized the role of the thymus and other lymphoid organs in the generation of different lymphocyte populations (64, 65). At about the same time, the UK immunologist, James Gowans (1924–), had shown that the lymphocyte population was able to recirculate through the body and enter the different tissue sites—an important and necessary feature for T lymphocytes which mediate cellular immunity and hence depend on cell–cell contact (66). The producers of antibodies had been identified earlier, namely in 1940 by the Swedish researcher, Astrid Fragaeus (1913–1997), as plasma cells (67, 68). Her work as well as that of the US immunologist, Max Cooper (1933–) then led to the revelation that plasma cells are derived from B lymphocytes which develop in the Bursa fabricii in birds and in the bone marrow in mammals (64, 65, 69).

Now the major cells of the acquired immune response had been identified and immunologists increasingly focused on their biological functions (Figure 5). Henry Claman (1930–2016) was probably the first to provide compelling evidence that T lymphocytes and B lymphocytes collaborate in the generation of antigen-specific antibodies (70). Av Mitchison (1928–) showed that antibodies were specific for the epitope (Landsteiner's small residues—the haptens) and T cells for the protein carrier (71). The establishment of T lymphocytes and B lymphocytes as responsible cells of acquired cellular and humoral immunity, respectively, and their collaboration in shaping an optimal immune response laid the basis for the golden age of cellular immunity.

Following the footsteps of the founders of immunology, the Australian borne researcher working in the US, George Mackaness (1922–2007), extensively studied immunity against intracellular bacteria. He discovered the cooperation between specific T lymphocytes and mononuclear phagocytes. In this setting, antigen specific T cells stimulate increased antibacterial activities in macrophages which thereby change from a habitat for the intracellular pathogens to the major effectors of cell-mediated immunity against the infection (72).

Transplantation biology and immunobiology converged when Peter Doherty and Rolf Zinkernagel demonstrated that MHC molecules were not only responsible for transplant rejection, but for T-cell recognition of any type of antigen. Antigen recognition by T lymphocytes, therefore, was MHC-restricted and transplant rejection was just one special case (73). Their breakthrough work, honored by the Nobel Prize in 1996, was based on antigen recognition by cytolytic T lymphocytes, which kill virus-infected cells (73, 74). Soon these cells were characterized phenotypically as CD8 T cells, which were MHC I-restricted. CD8 T cell counterparts, the CD4 T cells, were MHC II-restricted and shown to activate other cells of the immune system, notably B cells and macrophages by means of soluble factors, the cytokines. Activation of macrophages increases antibacterial activities, which in turn allows macrophages to control intracellular bacteria, such as the causative agent of tuberculosis. B cell activation leads to the production of antibodies of different isotypes. CD4 T cells were also found to help CD8 T cells become killer T cells. The first molecularly defined T cell cytokine was interleukin-2 (IL-2), which was originally described by the US immunologist, Kendall Smith (1933–) (75). His findings paved the way for the discovery of numerous humoral mediators of T cell immunity. With the identification of many other cytokines, the concept of T helper 1 (TH1) vs. T helper 2 (TH2) cells was developed by the Canadian immunologist Tim Mosmann (1949–) and the US immunologist Bob Coffman (1949–) (76). CD4 T cells of TH1 type contribute to the cellular immune response by activating killer T cells and macrophages. IL-2 was identified as the major mediator of killer T cell activation and interferon-γ (IFN-γ), which had already been described earlier as immune IFN was shown to be critical for macrophage activation. In contrast, TH2 cells produce IL-4 and other cytokines, which stimulate B lymphocytes to mature to antibody-producing plasma cells. Early on it was recognized that the immune response is highly regulated and notably that a well-functioning immune response need not only be activated to combat an intruder, but also needs to be downregulated once the intruder had been eliminated. This led to the concept of a highly regulated immune response involving specific T cells with suppressive functions to avoid collateral damage. Early attempts to explain this issue postulated suppressor T cells which, however, did not stand the test of time. The more refined concept of the better defined subsets of regulatory T cells, however, provided compelling evidence for specific T lymphocytes which not only control immune responses after elimination of invading pathogens, but also prevent autoimmunity and maintain homeostasis (77).

Although the biological functions of T lymphocytes were increasingly better understood, their antigen receptors remained elusive until the 1980s. By using monoclonal antibodies, US immunologists, Pippa Marrack (1945–) and John Kappler (1943–) (in the mouse system) (78), and Ellis Reinherz (1950–) and Stuart Schlossman (1935–) (in the human system) (79), were able to phenotypically identify antigen-specific receptors on T lymphocytes. This was the first hint for the existence of the antigen-specific T cell receptor (TCR). Soon thereafter, genes encoding TCR chains were cloned by Tak Mak (1946–) in Canada and Mark Davis (1952–) in the US (80, 81).

**Figure 6:**
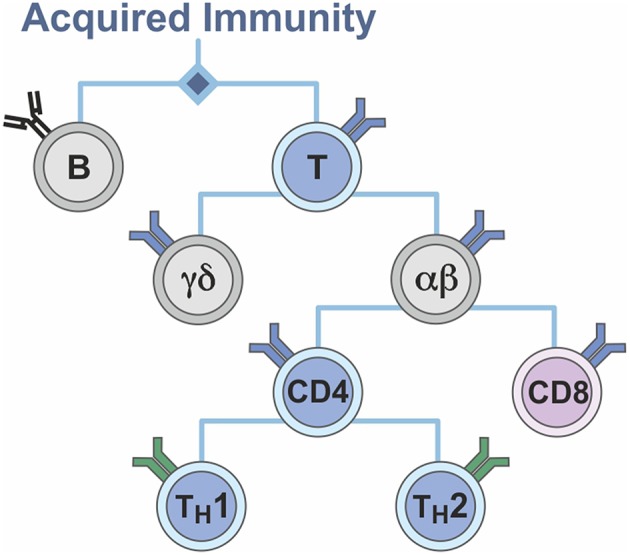
Binarity of the T cell system.

The T lymphocyte system can thus also be viewed as a binary system (Figure 6). Lymphocytes segregate into B and T cells; T cells segregate into MHC I- and MHC II-restricted T cells of CD4 or CD8 phenotype, respectively; CD4 T cells separate into TH1 and TH2 cells; the vast majority of T cells express a T cell receptor composed of an α and a β chain, but a second T cell population exists, which expresses a T cell receptor comprising a γ and a δ chain. Again, support was withdrawn for a dichotomous view, in favor of a complementary dualism (Figure 5).

### Recombination Generates Diversity

These important findings were preceded by the breakthrough discovery of the Japanese researcher, Susumu Tonogawa (1939–), then in Basel, Switzerland, who elucidated the mechanisms underlying the huge diversity of antibody specificities (82, 83). By then it was generally accepted: a single specific B cell was responsible for antibody production; diversity was generated prior to the first contact with antigen; a single B cell expresses a receptor with a unique specificity; contact with the homologous antigen stimulates selective expansion and differentiation of the specific B cell. Yet, one critical issue remained unsolved, namely that the number of possible antibody specificities exceeded the number of genes present in our body. The solution to this was identified by Tonegawa as the rearrangement of gene fragments. This recombination allows the generation of more than one million specificities which further increases numerically by additional mechanisms to up to some 10^9^ specificities. Tonegawa was honored with the Nobel Prize in 1987 “for the discovery of the genetic principle for generation of antibody diversity” (84). Principally, antigen diversity of the T cell receptor is based on similar genetic mechanisms.

### T-Cell Instruction by Antigen-Presenting Cells

In any case, the specificity of the acquired immune response and the multiple roles played by T cells more or less dominated immunobiology in the 1960s to 1990s. An influential researcher in the field of T cell immunology was Charles Janeway (1943–2003) from the US (85), who in a remarkable paper published in 1989 in the Proceedings of the Cold Spring Harbor Symposium, pointed to the widely underestimated role of the innate immune system (86). Prevailing opinion was that innate immune cells, notably macrophages and neutrophils, play an important effector role in host defense, under the guidance of T lymphocytes and their soluble products. Even though it was clear that T cells recognize antigens in the context of MHC presented on the surface of so-called antigen-presenting cells, these cells were viewed more as passive guides than active players. Janeway postulated the presence of pattern recognition receptors on antigen-presenting cells, which sense specific motifs of chemical products of bacteria and viruses and then instruct T cells about the different functions they should perform. Most compelling evidence for such an idea came from studies on the toll-like receptors (TLR) in mammals by the US geneticist Bruce Beutler (1957–), and in insects by the biochemist Jules Hoffmann (1941–) in France (87, 88). This led to the concept that different types of pathogens are sensed by pattern recognition receptors with specificity for microbe-associated molecular patterns. Beutler and Hoffmann jointly received the Nobel Prize in 2011 “for their discoveries concerning the activation of innate immunity” (89). The concept of sensing of microbial motifs (so-called pathogen-associated molecular patterns, PAMP) by innate receptors was soon broadened when similar mechanisms were found to be induced by host motifs (so-called danger associated molecular patterns, DAMP) which arise from insult to the host (90). In how far PAMP and DAMP influence immune tolerance by inducing danger associated non-self or self-signals to the induction of an acquired immune response remains a matter of controversial discussion (91, 92).

As early as the 1970s, the Canadian immunologist Ralph Steinman (1943–2011) at Rockefeller University, US, was engaged with defining the critical player in this concept: the dendritic cell (93). He demonstrated that dendritic cells are much more potent antigen presenters than macrophages, and that they are the major instructors of T cells regarding the type of pathogen they will encounter. Steinman was the third to be honored by the Nobel Prize 2011 “for his discovery of the dendritic cell and its role in adaptive immunity” (89). Sadly he could not accept the award in person because he passed away shortly before the ceremony. In conclusion, innate immunity plays a crucial role, from the beginning to the end of an immune response. In the beginning it acts via antigen-presenting cells, which not only stimulate antigen-specific T cells but also serve as instructors for the biological functions T cells have to perform. Toward the end, innate immunity takes care of effector functions, e.g., via professional phagocytes which eliminate invading pathogens.

Instruction of T cell functions strongly depends on cytokines, i.e., humoral factors. Thus, IL-12 induces TH1 cells whereas IL-4 directs TH2 cells. In fact, the first chemically defined cytokine was described by the US immunologist, Charles Dinarello (1943–) as a macrophage-derived product, which accordingly was later named IL-1 (94). IL-1 plays a role in the instruction of TH1 cells and serves as mediator of inflammation.

### From Serum Therapy to Checkpoint Control

B cells stood in the shadow of T lymphocytes during the 1970s. The discovery by the Argentinian researcher, Cesar Milstein (1927–2002), and the German researcher, Georges Köhler (1946–1995), both working in the UK, brought them back to center stage. In 1984, both shared the Nobel Prize “for the production of monoclonal antibodies” (58). Obviously, this discovery had major implications. First, it allowed the ultimate proof for the production of an antibody with single specificity by a single plasma cell and second, it paved the way for a new era of immunotherapy. As a short reminder, the concept of acquired immunity started with antibodies and was intrinsically intertwined with the concept of serum therapy, for which Behring received the Nobel Prize in 1901. Now the tools for more precise passive immunization had been put on the table. This led to the development of a number of monoclonal antibody-based therapies for infectious diseases; currently, the focus of monoclonal antibody therapy is on immunomodulation. Thus, cytokine-blocking monoclonal antibodies have been introduced in the treatment of chronic inflammatory diseases. Most notable are Infliximab and Adalimumab, which block the critical cytokine tumor necrosis factor-α (TNF-α) in Crohn's disease and rheumatoid arthritis, respectively (95, 96). A second important target of therapeutic monoclonal antibodies are surface-expressed molecules such as CD20 on B lymphocytes, which can be harnessed for treatment of non-Hodgkin's lymphoma such as Rituximab (97).

A major recent breakthrough has been the discovery of monoclonal antibodies which block checkpoint control. What does this mean? Regulation of T cell activity is not only a matter of cytokines but also of costimulatory molecules, which in addition to TCR recognition of antigen plus MHC as first signal, provide a second signal for T cells in stimulating their effector functions. Eventually, the immune response needs to be dampened. Once it has completed its task, e.g., after the elimination of an infectious agent, it needs to be tuned down to avoid or at least minimize collateral damage. Surface-expressed inhibitory molecules include CTLA-4 and PD-1 on T cells and their counterparts B7 and PD-L1 on antigen-presenting cells (98, 99). These counterparts are also expressed on many tumor cells, which block attack by killer T cells. Blockade of checkpoint control improves T cell responses and thereby allows elimination of certain tumor cells. This finding led to next-generation immunotherapies for certain cancers including metastatic melanomas and non-small cell lung carcinomas. The highly promising checkpoint blockade for cancer therapy was honored by the Nobel Prize 2018 to the US immunologist Jim Allison (1948–) and Japanese immunologist Tasuku Honjo (1942–) “for their discovery of cancer therapy by inhibition of negative immune regulation (5).”

## Short Recap and Outlook

**Figure 7:**
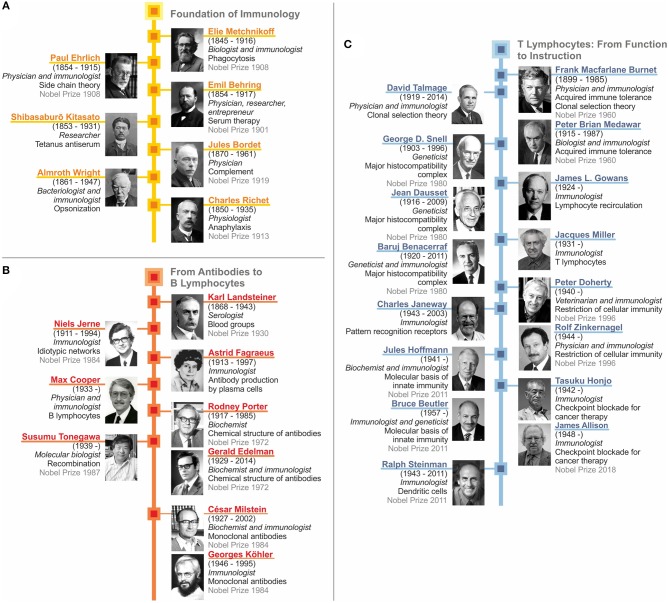
Recap of immunology: from serum therapy to checkpoint control. **(A)** Foundation of immunology. **(B)** From antibodies to B lymphocytes. **(C)** T lymphocytes: from function to instruction.

As we have seen, immunology as a scientific discipline was kick-started by two seminal discoveries: First, the role of phagocytosis performed by cells and second, the neutralization of bacterial toxins by antibodies. This led to the concept of dichotomous roles of antigen-unspecific innate immunity mediated by cells and antigen-specific acquired immunity mediated by humoral factors. This dichotomous concept converged with the identification of complement and opsonization, which linked innate and acquired immunity. Major early contributors are depicted in Figure 7A. The intermediate stage includes the discovery of different forms of clinical hypersensitivity emphasizing that the immune system also embodies detrimental functions. In parallel, immunochemistry reached its climax with the elucidation of the crystal structure of antibodies. Then immunobiology took over with the identification of lymphocytes and their segregation into antibody-producing B cells and plasma cells, as well as T cells, which function as central regulators of immunity (Figures 7B,C). TH cells were shown to control B lymphocytes, professional phagocytes and cytolytic T cells. Finally, this dysbalanced perception of acquired immunity dominating innate immunity was rectified by our increasing understanding of how antigen-presenting cells instruct the acquired immune response (Figure 7C). Today sufficient knowledge has been accumulated in immunology to devise sophisticated therapeutic approaches, such as checkpoint control for cancer treatment. Yet, in both basic and applied immunology, sufficient challenges persist which guarantee that our discipline will remain as vital as ever.

Importantly, the immune apparatus is increasingly seen as a highly diffuse organ comprising not only bone marrow, thymus and spleen, but also lymph nodes and lymphoid follicles which are spread throughout the body and interconnected by circulating leukocytes and soluble mediators. Accordingly, immune cells are imprinted by their organ of residence to adjust to the special regional needs. Reciprocally, immune cells impact on the tissue of their main residence. Moreover, our microbiome is increasingly viewed as a human organ vital to health and disease and tightly intertwined with the immune system. As a corollary, dysfunctions of regional immune responses underlie many organ-specific diseases. Future immunology will have to take into account an integrated view on these crosstalks at all levels from organs to tissues to cells to molecules. The enormous advances in high-throughput multi-omics technologies and bioinformatics allow studies on multiple levels of the immune response thus providing a wealth of data which will ultimately result in the construction of molecular multi-networks of the immune response under physiologic and pathologic conditions. Ultimately, this system biology approach will provide a far more comprehensive perspective of immunology which will generate new concepts for prevention and treatment of diseases that are refractory to current intervention strategies due to dysfunctional, insufficient or subverted immunity. Paul Ehrlich's dream of “magic bullets” will take a step closer to reality by the immunology of the future.

## Author Contributions

The author confirms being the sole contributor of this work and has approved it for publication.

### Conflict of Interest Statement

The author declares that the research was conducted in the absence of any commercial or financial relationships that could be construed as a potential conflict of interest.
