# Corrigendum: Immunology's Coming of Age

**DOI:** 10.3389/fimmu.2019.01214

**Published:** 2019-06-06

**Authors:** Stefan H. E. Kaufmann

**Affiliations:** ^1^Department of Immunology, Max Planck Institute for Infection Biology, Berlin, Germany; ^2^Hagler Institute for Advanced Study, Texas A&M University, College Station, TX, United States

**Keywords:** antibody, cytokine, dendritic cell, immunology, lymphocyte, macrophage, phagocytosis, recombination

In the original article, there was an error. The birth dates used in some of the citations were incorrect.

Corrections have been made to **ACT I: The Foundation of Immunology**, paragraph one:

“Immunology emerged as an academic discipline in its own right out of the fertile soil of medical microbiology (6). The discoveries of Louis Pasteur (1822–1895), which confirmed and completed the germ theory of infectious diseases as well as Robert Koch's (1843–1910) meticulous studies on the etiology of infectious diseases, notably tuberculosis, raised a question of fundamental importance: Is the host a helpless prey of pathogenic microbes or is it equipped with an efficient defense mechanism to combat its invaders? Both Pasteur and Koch favored the notion that the host was defenseless. However it was Metchnikoff, at the Pasteur Institute in Paris since 1888, who earlier discovered the critical role of phagocytosis and intracellular killing in host defense (1), and it was Behring and Ehrlich, young independent researchers at Koch's institute for Infectious Diseases in Berlin, who identified antibodies as crucial counterparts to the toxic activities of bacteria (1, 2). We now know that the outcome of infection depends on close interactions between pathogen and host factors, probably best described by the term infection biology.”

**Act II: Immunochemistry and Clinical Immunology**, subsection **Immunochemistry**:

“During the first half of the twentieth century, immunologists focused on clinical observations and even more on immunochemistry, which could build on a much broader armamentarium of technical tools. Immunochemistry found its culmination in the discovery of the chemical structure of antibodies (Figure 4). This was accomplished independently by the British chemist, Rodney Porter (1917–1985), and the US chemist, Gerald Edelman (1929–2014), in the late 1950s to early 1960s (33, 34). Their work was honored by the Nobel Prize in 1972 (35). The Austrian Karl Landsteiner (1868–1943), first working in Europe and since 1923 in the US, developed the carrier hapten concept by coupling small aromatic molecules to proteins (36). He showed that the small residue—the hapten—is recognized by antibodies, and therefore serves as epitope, and that the protein serves as carrier to provide the immunogenicity needed for successful stimulation of an antibody response (37, 38). Since the studies of Jacques Miller (1931–), Henry Claman (1930–2016) and others, we know that the antibody response involves B lymphocytes for the recognition of the hapten and T lymphocytes for the recognition of the carrier.”

**Act II: Immunochemistry and Clinical Immunology**, subsection **Hypersensitivity Reactions**, paragraph one:

“Landsteiner is probably best known for the discovery of the ABO major blood group system (39). Working at the time in Vienna, he found that mixing blood of two different individuals resulted in clumping of red blood cells. Based on this finding, he developed a technique for the serologic differentiation of erythrocytes, which allowed him to identify the different blood groups of the ABO system. This discovery was honored by the Nobel Prize in 1930 (40). Ten years later, and together with Alexander Wiener (1907–1976), Landsteiner discovered a second important blood group, called Rhesus (Rh), named after their original discovery with erythrocytes in Rhesus monkeys (41, 42).”

**Act III: The rise of immunobiology**, subsection **Transplantation Biology**:

“The 1950s to 1960s witnessed a marked shift in priorities from immunochemistry to immunobiology (Figure 5). In fact, studies on transplant rejection preceded and prepared the ground for immunobiology. The US geneticist George Snell (1903–1996), based on his studies with inbred mouse strains, elegantly demonstrated that distinct genes within the major histocompatibility complex (MHC) were responsible for transplant rejection (51). The French clinician, Jean Dausset (1916–2009), discovered the human MHC, also named human leukocyte antigen (HLA), on the basis of family studies (51). A somewhat more direct link to immunobiology was provided by the Venezuelan-born US scientist, Baruj Benacerraf (1920–2011), who identified the immune response genes within the MHC locus (51). In 1980, Snell, Dausset and Benacerraf were honored by the Nobel Prize “for their discoveries concerning genetically determined structures on the cell surface that regulate immunological reactions” (52). Later the Australian researcher, Peter Doherty (1940–), and the Swiss researcher, Rolf Zinkernagel (1944–), would broaden this perspective by showing that the MHC is crucial for antigen recognition by T lymphocytes, the cells that would become the dominant research target in the second half of the twentieth century.”

Additionally, the birth date provided for George D. Snell in Figure 7C, should be corrected from “1903–1966” to “1903–1996”. The corrected Figure 7C appears below.

**Figure 7:**
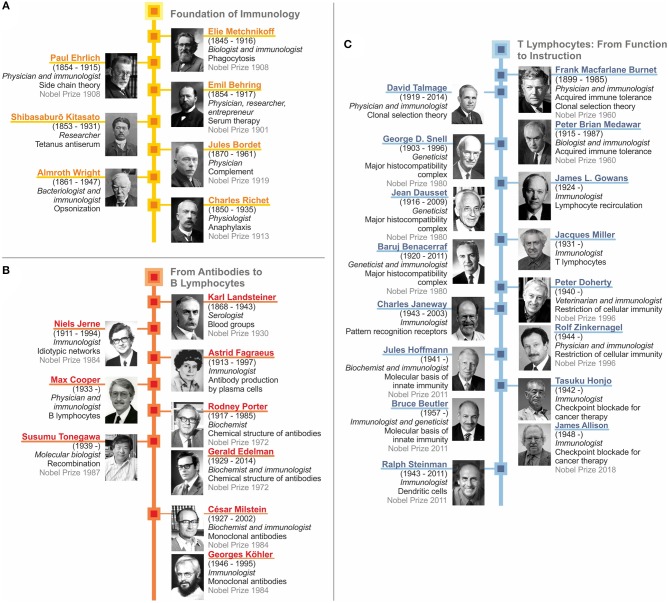
Recap of immunology: from serum therapy to checkpoint control. **(A)** Foundation of immunology. **(B)** From antibodies to B lymphocytes. **(C)** T lymphocytes: from function to instruction.

The authors apologize for this error and state that this does not change the scientific conclusions of the article in any way. The original article has been updated.

